# Acalculous cholecystitis– an imaging and therapeutic update

**DOI:** 10.1007/s00261-024-04691-0

**Published:** 2024-12-16

**Authors:** Matthew A Morgan, Daniel M DePietro, Debra S Whorms, Austin R Pantel, Dhakshinamoorthy Ganeshan, Inessa A Goldman, Julie Yang, Rachita Khot

**Affiliations:** 1 University of Pennsylvania Health System, Philadelphia, USA; 2 The University of Texas MD Anderson Cancer Center, Houston, USA; 3 Rutgers New Jersey Medical School, Newark, USA; 4 Icahn School of Medicine at Mount Sinai, New York, USA; 5 University of Virginia Health, Charlottesville, USA

**Keywords:** Acalculous cholecystitis, Cholecystitis, Gallbladder, Radiology

## Abstract

In this review, we highlight current understanding of the pathogenesis of acalculous cholecystitis, as well as its key imaging and clinical features. We also review what happens after a diagnosis and outline current interventional methods.

**Supplementary Information:**

The online version contains supplementary material available at 10.1007/s00261-024-04691-0.

## Introduction

Acalculous cholecystitis is an important and often under-appreciated source of morbidity (and potential mortality), especially in critically ill patients. The clinical and imaging findings are often nonspecific and an index of suspicion from both the clinical team and the radiologist are needed to avoid a delay in diagnosis. In this review, we highlight current understanding of the pathogenesis of acalculous cholecystitis, as well as its key imaging and clinical features. We also review what happens after a diagnosis and outline current interventional methods.

### Pathogenesis

The development of gallbladder inflammation in the absence of gallstones defines acute acalculous cholecystitis (AAC) [1]. In contrast to acute calculous/gallstone cholecystitis (ACC), which occurs predominantly in younger women, acalculous cholecystitis is reported more commonly in elderly men. Furthermore, although this condition is less common in children compared to adults, acalculous cholecystitis accounts for over 50 to 70% cholecystitis occurring in the pediatric population [2]. A chronic form of acalculous cholecystitis (CAC) has also been described, which is defined as acalculous cholecystitis lasting for three months.

The exact pathogenesis of acute acalculous cholecystitis is not entirely clear but reported to be multifactorial [3, 4]. Obstruction of the cystic duct, which is the precipitating factor in ACC, is not necessary. Instead, AAC is precipitated by critical systemic disease, likely a combination of ischemic-perfusion injury and cholestasis which results in mucosal injury. Activation of phospholipase A2 and superoxide dismutase, as well as increase in the content of lipid peroxides may play a role. The mucosal injury likely permits secondary bacterial invasion from enteric organisms such as *E. coli*, *E faecalis*, *Klebsiella spp.*, *Pseudomonas spp.*, *Proteus spp.*, and *B. fragilis*. [3, 5].

On histopathologic evaluation of gallbladders resected for AAC, severe vascular congestion and edematous changes are seen. Gallbladder wall gangrene was noted in up to 50% of the AAC specimens, and perforation in over 10%, compatible with the significantly increased mortality (>30%) associated with this condition [1, 6]. The histopathological features of AAC are not specific and may overlap with those seen in acute calculous cholecystitis. However, there is a higher prevalence and more extensive bile duct infiltration, epithelial degeneration, necrotic changes in AAC [7]. Furthermore, significant differences have been reported in the microangiography of AAC compared to ACC. AAC is associated with poor and irregular capillary filling, in contrast to the normal capillary filling and dilated arterioles seen in ACC showing higher prevalence of arterial dilatation and normal capillary filling [8]. Gallbladder wall hypertrophy is a feature that can be seen in patients with CAC.

AAC is associated with critically ill patients who have been hospitalized, including patients with acute severe infections/sepsis, major surgeries, severe trauma, burns, vasculitis, mechanical ventilation, and in those who or receiving cytotoxic drugs or total parenteral nutrition. [1, 5, 9]. Immunosuppressed patients are also at higher risk, such as those with acute leukemia, bone marrow transplant recipients, and patients with Acquired Immune Deficiency Syndrome [1].

Despite this association and proposed pathogenesis, acalculous cholecystitis has also been reported in the outpatient population with chronic medical disorders including those with diabetes, hypertension, end stage renal disorders and heart failure [9]. Ischemic/reperfusion injury of the gallbladder seems to be the common factor between the groups.

A related group of patients with mechanical obstruction of the cystic duct unrelated to gallstones such as extrinsic compression from porta hepatis adenopathy or congenital conditions such as choledochal cysts or stenosis of the cystic duct may also predispose to AAC, especially in the pediatric population. The pathogenesis is like ACC, but since the precipitating factor is not gallstones, technically, it would be included in AAC.

### Clinical features and tokyo guidelines

In general, clinical presentation of AAC mimics that of ACC. Patients are typically acutely ill and may develop fever, tachycardia, hypotension, and abdominal pain [1, 3–5]. However, it is extremely important to note that critically ill patients -- such as those in the intensive care unit -- may not present with any abdominal symptoms at all, or the signs and symptoms may be subsumed within a larger critical condition. These patients may also be intubated/obtunded. Since the presentation may be masked, an increased level of suspicion for AAC is necessary in this group of patients [1, 3].

Clinical examination may reveal a distended gallbladder or sharp tenderness/pain to manual pressure in the right upper quadrant with inspiration (positive Murphy sign). Laboratory tests typically show increased levels of acute inflammatory markers such as leukocytosis, although normal levels may be misleading in immunosuppressed patients.

Because of the often-nonspecific clinical features of cholecystitis, a set of guidelines (The Tokyo Guidelines, most recently revised in 2018 (TG18)) have been developed and are widely accepted for diagnosis and management of acute cholecystitis (AC), though no specific distinction is made regarding AAC.

The current guidelines on diagnostic criteria were shown in a multicenter validation study to have a sensitivity of 91.2% and specificity of 96.9% [12, 13].

The criteria assess:

1) Local signs of inflammation

2) Systemic signs of inflammation

3) Imaging findings

A definite diagnosis is established if there are positive findings from all three categories. While the sensitivity and specificity of hepatobiliary scintigraphy is superior to US [12], TG recommends US as the first choice of imaging due to its low cost and ease of use while CT or MRI is recommended if a definite diagnosis cannot be provided. The role of CT is especially vital in establishing a diagnosis of emphysematous or gangrenous cholecystitis, which has a higher incidence in AAC and since many of these patients will have a negative sonographic Murphy sign [14].

The TG also establish severity grading from Grade I to III, which has been demonstrated in validation studies to correlate with several outcome variables including hospital stay, conversion from laparoscopic to open surgery, complication rate, and overall 30-day mortality [12].

Grade I (or mild) AAC has no associated organ dysfunction, grade II (or moderate) AC shows marked local inflammation without organ dysfunction, while grade III (or severe) AC shows signs of organ system dysfunction. For treatment, TG18 recommends management based on disease severity as well as a patient’s general status and comorbidities as predicted by the Charlson comorbidity index (CCI) and the American Society of Anesthesiologist physical status classification (ASA-PS). For patients with grade I and favorable surgical risk, early cholecystectomy is the first line treatment, while treatment in grade II should include medical treatment to reduce inflammation followed by early cholecystectomy or drainage with delayed elective laparoscopic cholecystectomy depending on surgical risk and surgeon and facility capabilities. The recommendations for Grade III AC were revised in TG18, with the guideline now recommending that early cholecystectomy be performed at advanced centers in patients with favorable organ system failure—defined as rapidly reversible renal and cardiac damage—and without negative predictive factors including jaundice, neurologic dysfunction and respiratory dysfunction. Other patients with Grade III AC not meeting these strict criteria are recommended for urgent gallbladder drainage followed by observation or delayed cholecystectomy for those in good performance status [15].

However, while some studies have demonstrated similar prognosis of AAC compared to ACC adjusting for patient comorbidities [14, 16], there are significant differences in clinical course and treatment considerations between AAC and ACC that are not specifically addressed in TG18. For example, the frequency of recurrence after nonsurgical management is substantially lower in AAC compared to ACC [14] and interval cholecystectomy might not beneficial [17]. Only a single retrospective study is available specifically assessing validity of TG on patients with AAC. This showed similar distribution of severity in a cohort of 432 patients with AAC compared to patients with AC of any cause, with 38.6%, 46.8% and 14.6% of patients classified as Grade I, II, III respectively [15]. This study showed that TG18 was beneficial in management of Grade I and II AAC, however for patients with Grade III AAC there was no significant difference in outcomes for those who were treated according to TG18 with immediate cholecystostomy/drainage compared to those whose management deviated from the guideline with upfront cholecystectomy [15]. Likely owing to the higher frequency of critically ill patients in patients with AAC, less than 10% of the cohort with severe AAC would have qualified for cholecystectomy under TG18 [15]. Overall, these highlight potential pitfalls of TG18 in management of AAC, specifically in patients with severe/grade III AC.

### Imaging features

Imaging plays an important role in diagnosis of AAC, severity assessment, ruling out other conditions that may present with similar symptoms, identification of complications, and guiding treatment strategy. There are multiple imaging modalities available to image the gallbladder, including ultrasound (US), computed tomography (CT), magnetic resonance imaging (MRI), and hepatobiliary iminodiacetic acid (HIDA) scans. Depending on the stage and severity of the inflammation, the imaging findings in AAC varies.

### Ultrasound

Ultrasound (US) is the preferred first-line imaging modality of choice for AAC due to its wide availability, cost-effectiveness, and lack of ionizing radiation. Its portability makes it useful in critical care settings, allowing for bedside assessments [18]. US enables direct visualization of the gallbladder and surrounding structures, providing essential diagnostic information. However, its sensitivity ranges from 30 to 92%, and specificity from 89 to 100%, both of which can vary significantly depending on operator skill, patient body habitus, and the clinical context [1].

**Fig. 1:**
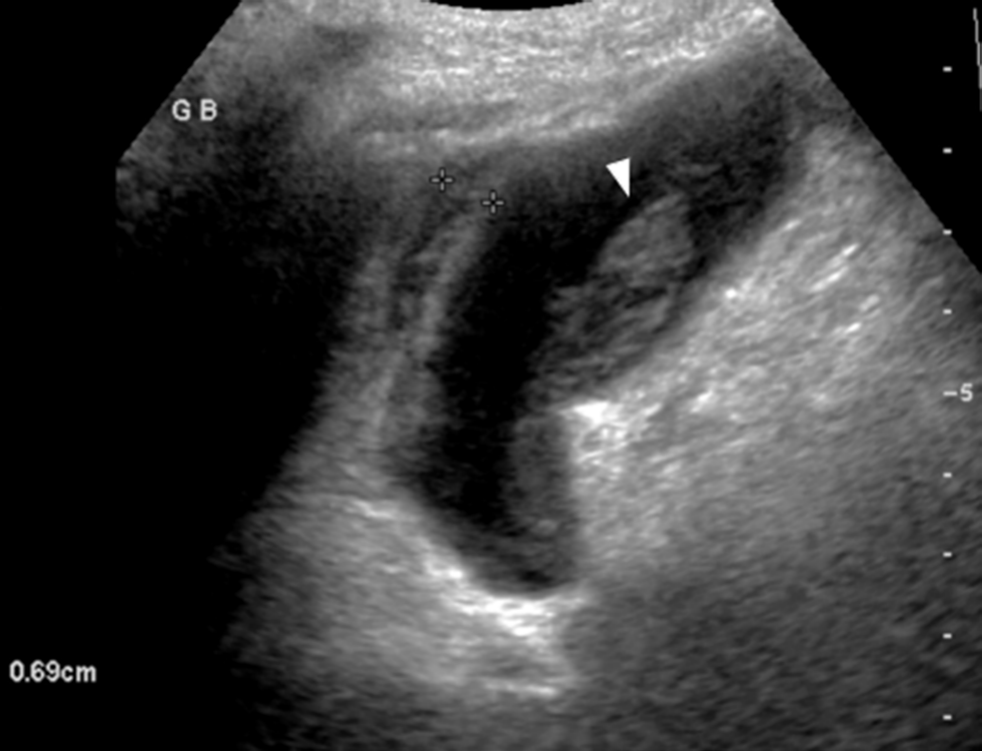
Acalculous acute cholecystitis in a critically ill 82-year-old female with altered mental status, increasing leukocytosis and right upper quadrant tenderness.Long axis ultrasound image of the gallbladder demonstrates thick and striated wall (between calipers), and echogenic sludge (arrowhead). No echogenic stone with posterior shadowing present

US examination of the gallbladder is performed using a curved 3 to 5 MHz transducer. The patient is positioned supine or in a left lateral decubitus with the right arm raised above the head for optimal visualization. Ultrasound often provides superior spatial resolution of gallbladder contents and wall compared to CT, allowing for the detailed assessment of key features of inflammation and ischemia. These include gallbladder wall thickening (> 3 mm), wall striations caused by edema, pericholecystic fluid, echogenic bile, mucosal irregularity, and, in some cases, frank perforation (Table 1). Identifying gallbladder distension, with a transverse diameter of >5 cm and length > 8 cm, is also important. (Fig. 1) [19]. Contrast-enhanced US in acute cholecystitis demonstrates increased enhancement of the wall before enhancement of the adjacent liver parenchyma during the arterial phase. The wall thickening is well seen during the late phase [20].

**Table 1:** Imaging criteria for diagnosing Acute acalculous cholecystitis on US and CT ^[1]^

Modality	Major Criteria	Minor criteria
	Gallbladder wall thickening > 3 mm	Gallbladder distention (> 8-cm longitudinal or 5-cm transverse diameter)
	Striated gallbladder	Echogenic bile
Ultrasound*	Sonographic Murphy sign	
	Pericholecystic fluid	
	Mucosal sloughing	
	Intramural gas	
	Gallbladder wall thickening > 3 mm	Gallbladder distention (> 5 cm in transverse diameter)
	Subserosal halo sign	High-attenuation bile
CT*	Pericholecystic infiltration of fat	
	Pericholecystic fluid	
	Mucosal sloughing	
	Intramural gas	

* Either two major criteria, or one major criterion and two minor criteria satisfy the diagnosis of AAC

A “sonographic” Murphy sign characterized by sharp pain or tenderness when pressure is applied by the ultrasound probe, may indicate AAC, though it can be unreliable in patients with altered mental status or those who have received pain medication [21].

### Nuclear medicine (HIDA)

Gallbladder scintigraphy (often referred to as a “HIDA scan”), images gallbladder physiology, providing complementary diagnostic data to other imaging modalities. It has a sensitivity and specificity greater than that of US, but since it is not as readily available as US, it is used as a second line study.

**Fig. 2:**
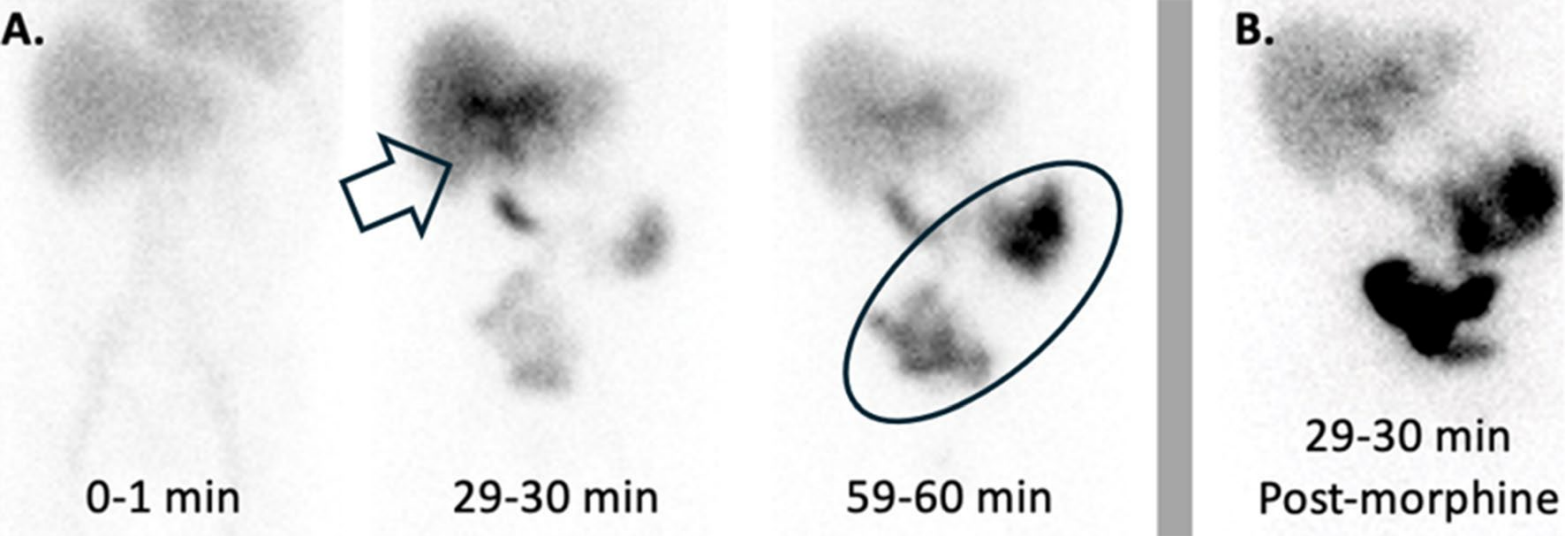
A 47-year-old male with nonischemic cardiomyopathy status post recent left ventricular assist device (LVAD) placement presents with right upper quadrant pain. An ultrasound demonstrated possible acalculous cholecystitis and gallbladder scintigraphy was obtained for further evaluation. Three representative images are shown in (**A**) following the intravenous administration of 5.6 mCi Tc-99 m mebrofenin, with the images labelled with the acquisition time. The open arrow in the second image shows the tracer concentrating in the central bile ducts, extending down through the common bile duct, and then beginning to enter the small bowel. On the next image (oval), most of the tracer has now moved from the bile ducts with an increasing amount in the small bowel. The gallbladder was not visualized on the images in A, so morphine sulfate was given (0.04 mg/kg) and imaging were obtained for an additional 30 min. A representative imaging in (**B**) demonstrates nonvisualization of the gallbladder after morphine administration, consistent with acute cholecystitis (acalculous given the lack of stones on ultrasound)

The procedure involves administering a radioactive tracer, such as 99mTc-disofenin or 99mTc-mebrofenin and imaging its execration through the biliary system. Historically, these scans were named after the original tracer, hepatoiminodiacetic acid (HIDA) [22]. Following injection, the abdomen is imaged for approximately 60 min. If the gallbladder fills with the tracer, this indicates a patent cystic duct, effectively ruling out acute cholecystitis, and the study may be concluded. However, if the gallbladder is not visualized, delayed imaging may be obtained in 3–4 h or following morphine administration with an additional imaging at 30 to 60 min. Morphine induces constriction of the sphincter of Oddi, creating backpressure of radioactive bile and, if the cystic duct is patent, filling the gallbladder. Failure to visualize the gallbladder even after these steps confirms the diagnosis of acute cholecystitis (Fig. 2).

The diagnostic utility of gallbladder scintigraphy applies to both calculous and acalculous cholecystitis. In calculous cholecystitis, duct obstruction by a stone directly correlates with non-visualization of the gallbladder. Although there is no obstructing stone in acalculous cholecystitis, cystic duct obstruction may be due to other factors such as inspissated bile, cellular debris, or edema, which impede duct patency. However, around 25% of patients with acute acalculous cholecystitis have a patent cystic duct, which may lead to false-negative results. While this reduces the sensitivity of gallbladder scintigraphy to approximately 70%, it remains a highly specific test [22, 24]. In cases where a false negative is suspected, further investigation with a radiolabeled 111-Indium white blood cell scan may be considered [25].

### CT

A CT scan is performed when ultrasound findings are equivocal or further assessment of the gallbladder and the adjacent structures is required. The CT may also be acquired first when searching for a different pathology and findings of AAC are discovered incidentally. With a high sensitivity and specificity of approximately 95%, CT can identify complications such as perforation, pericholecystic abscess, peritonitis, or involvement of neighboring structures [18]. It helps differentiate AAC from other conditions that may present with similar symptoms, such as pancreatitis or hepatitis. Despite these advantages, in order to acquire the CT, the patient must be transported to the CT scanner, which can be a limiting factor in critically ill patients [18, 19].

**Fig. 3:**
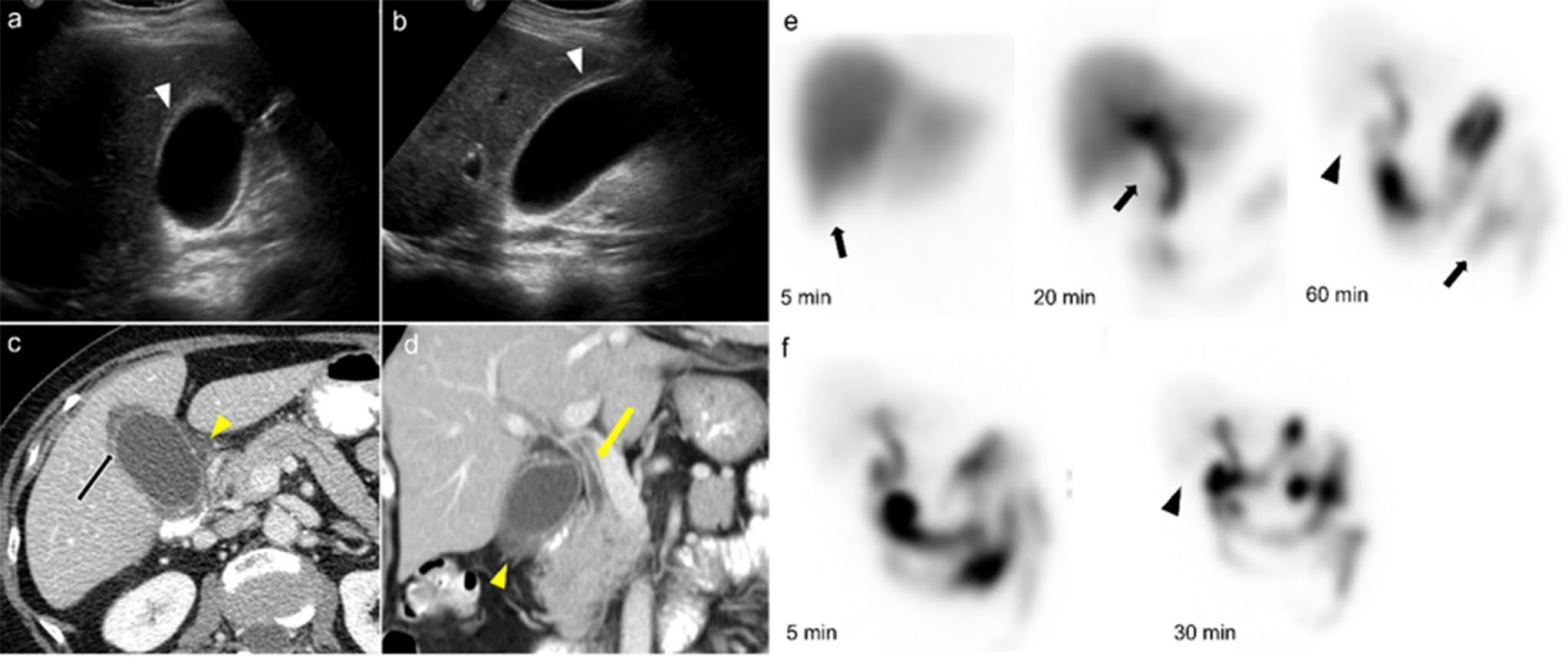
79-year-old female with right upper quadrant colicky postprandial pain. Ultrasound axial (image **a**) and long axis (image **b**) demonstrates wall thickening and hypoechogenicity in the wall due to edema (arrowheads). CT and scintigraphy scans were performed after the ultrasound. CT axial (image **c**) and coronal view (image **d**) demonstrates wall thickening (arrowhead), mucosal hyperenhancement (arrow), pericholecystic stranding (yellow arrowhead) and extrahepatic bile duct wall thickening and increased enhancement (yellow arrow) secondary to inflammation. The scintigraphy scan (image e) anterior image shows prompt uptake of the radiotracer by the liver (arrow), prompt excretion into the intrahepatic ducts, common bile duct by 20 min (arrow) and normal transit into the small bowel (arrow). There is no visualization of the gallbladder by 60 min (arrowhead). 2 mg Morphine was given intravenously. 30 min after the Morphine administration, there is no gallbladder visualization (arrowhead, f)

The CT features of AAC are analogous to US, including gallbladder wall thickening (> 3 mm) with edema, pericholecystic fat stranding, pericholecystic free fluid, hyperdense bile, and gallbladder distension [19, 26]. In contrast-enhanced CT, mucosal hyperenhancement and adjacent hyperemic liver parenchyma are evident [21]. The extrahepatic bile duct involvement can be seen as wall thickening of the bile duct (Fig. 3) [27].

### MRI

MRI provides an excellent soft tissue contrast, aiding in the detailed characterization of gallbladder inflammation and differentiation between different gallbladder conditions. Like CT, it also is better than ultrasound at providing alternative diagnoses.

MRI has a sensitivity of 95% and specificity of 69% in the evaluation of gallbladder abnormalities [18]. Despite its high diagnostic accuracy, the applicability of MRI is restricted by the patient’s ability to cooperate during imaging, often challenging in the context of severe illness and often resulting in suboptimal diagnostic image quality [26]. Therefore, its use is limited to a supporting role for patients in whom US, CT and HIDA are equivocal or if management is likely to change in response to other findings in the abdomen. MRI findings are analogous to those seen in US and CT: gallbladder wall thickening, dilatation of the gallbladder, subserosal edema, and pericholecystic fluid in uncomplicated AAC [26–28]. The utility of hepatobiliary contrast agents such as gadoxetate in the setting of AAC is not well studied. Magnetic resonance cholangiopancreatography (MRCP) is often helpful to exclude concurrent choledocholithiasis [29, 30].

### Imaging complications of AAC

AAC is prone to complication and is associated with a higher incidence of complications compared to ACC. Gangrenous cholecystitis, in particular, is significantly higher in AAC compared to ACC (31.2 vs. 5.6%) [14] and puts the patients at risk for perforation. Perforation alone is seen in 10–20% of cases with AAC and increases the risk of pericholecystic abscess, cholecystoenteric fistula, and peritonitis [26, 31]. Other rare complications include emphysematous and hemorrhagic cholecystitis. There is also a 5-fold higher risk of bacteremia in AAC compared to ACC [32].

**Fig. 4:**
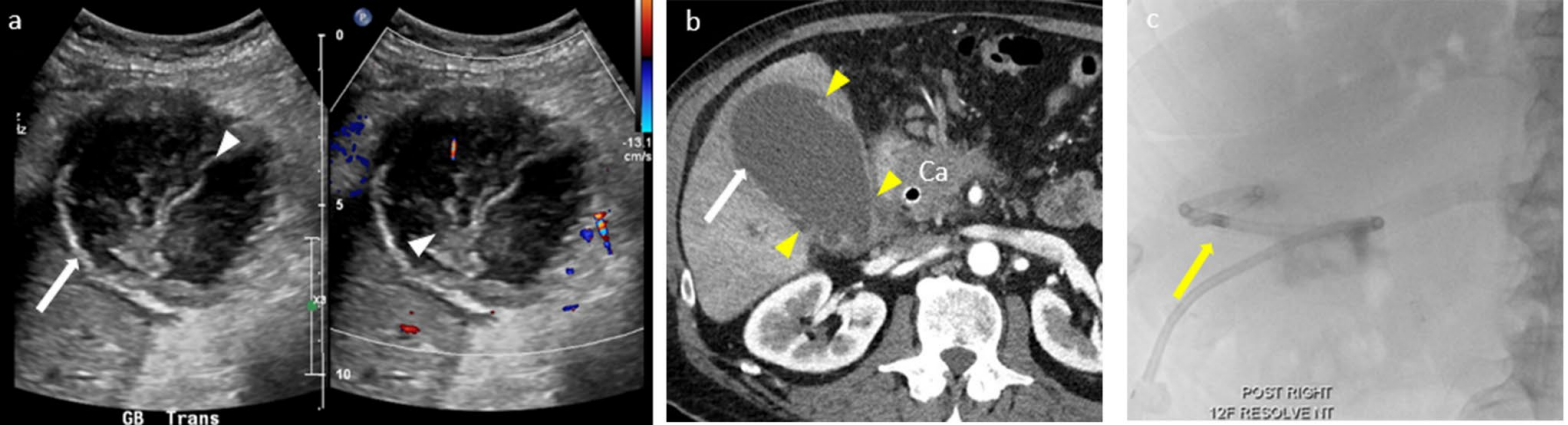
Gangrenous cholecystitis in a 61-year-old male with pancreatic cancer. Ultrasound (image **a**) demonstrates dilated gallbladder (arrow) with floating linear echogenic intraluminal membranes from sloughed mucosa (arrowheads). Contrast enhanced CT axial image (image **b**) shows dilated gallbladder (arrow), multiple focal wall irregularities and decreased enhancement due to sloughed mucosa and discontinuity of the wall (yellow arrowheads). A cholecystostomy tube was placed for management (image **c**, yellow arrow))

**Fig. 5:**
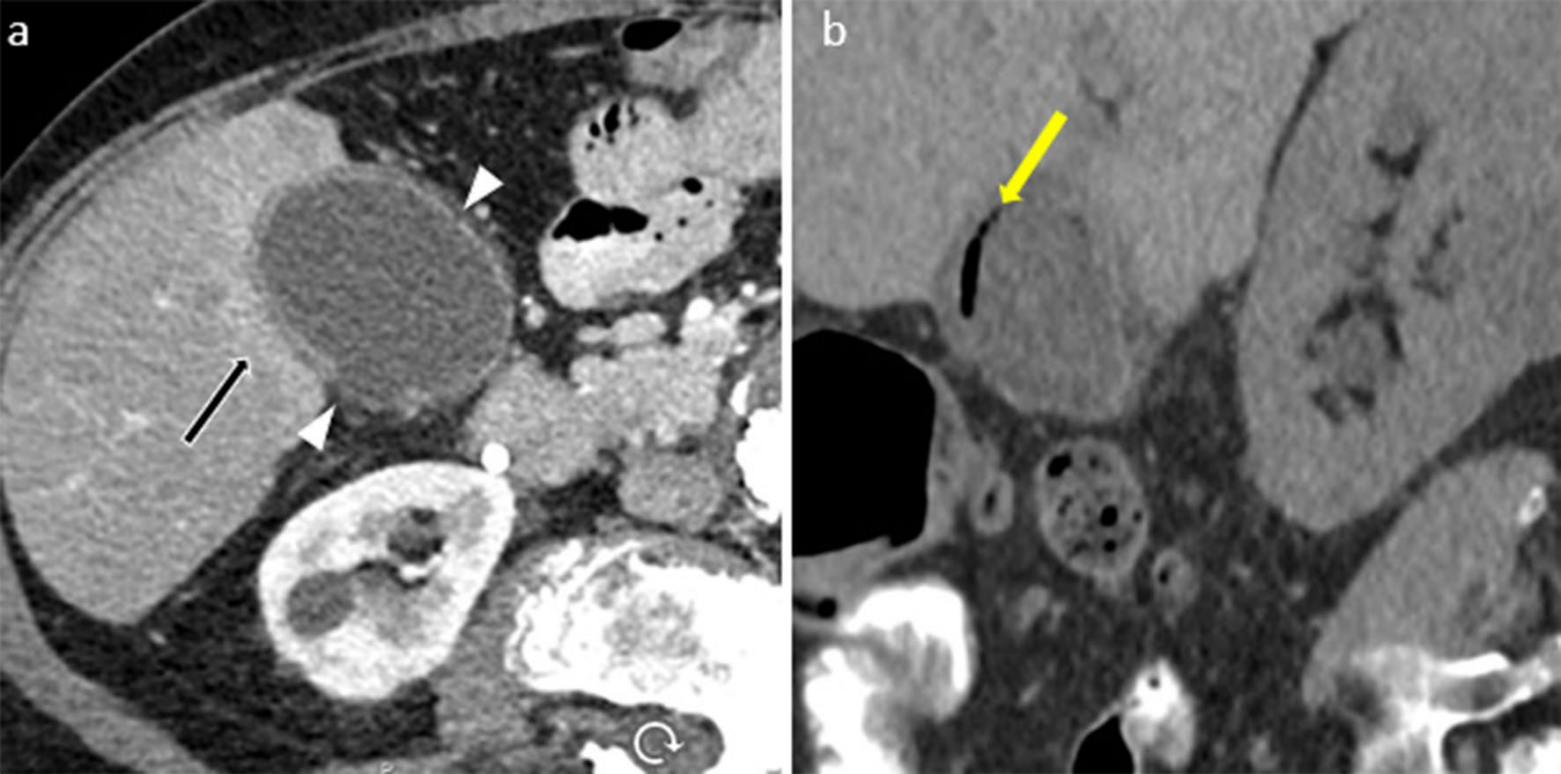
Gangrenous cholecystitis in a 68-year-old female with fever and diffuse abdominal pain post sigmoidectomy and colostomy. Contrast enhanced CT axial views (image **a**) demonstrates wall thickening and focal areas of poor or lack of gallbladder enhancement (arrowheads) with pericholecystic stranding, and increased enhancement of the adjacent liver parenchyma (arrow). The findings were complicated by development of gas in the wall (image **b**, yellow arrow) after 7 days of gangrenous cholecystitis diagnosis

**Fig. 6:**
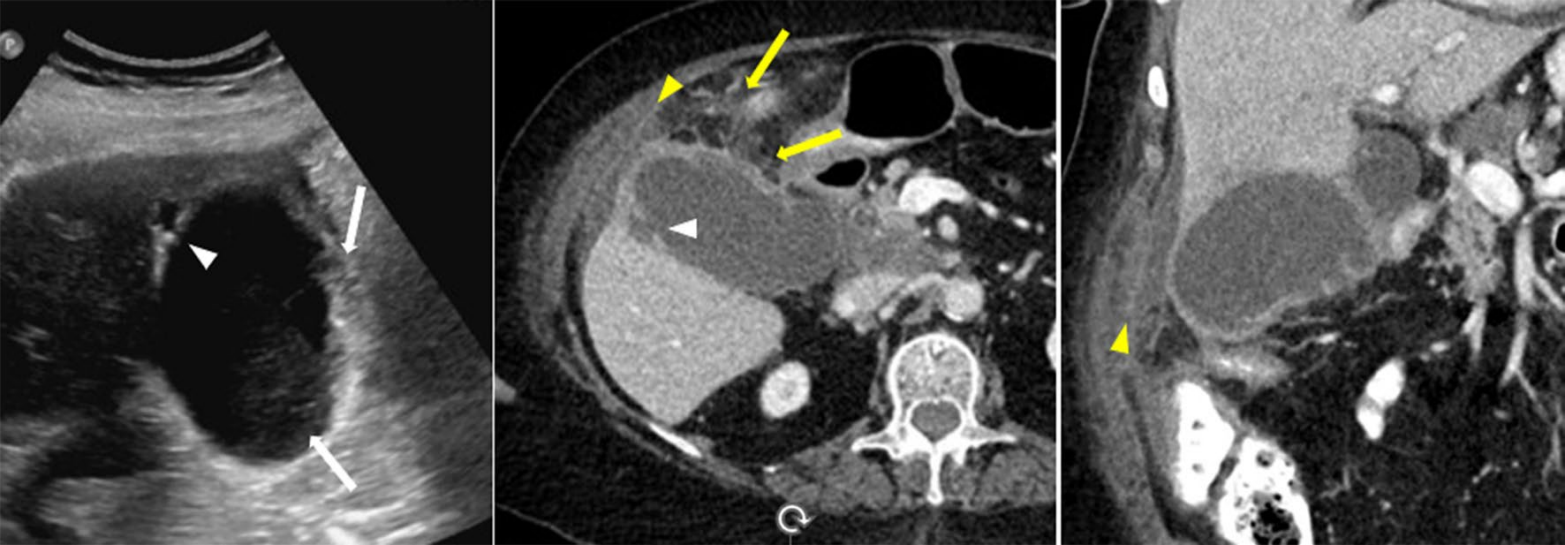
Gangrenous cholecystitis with perforation and peritonitis in a 91-year-old female with history of hypertension, presents with two weeks of right hemi-abdominal pain to the emergency room. Blood work showed, elevated white blood cell count, and elevation of the alkaline phosphatase. US was performed which demonstrates defect in the wall (arrowhead) in addition to the sludge and sloughed mucosa (arrows). Contrast enhanced CT confirms the finding of perforation (arrowhead). There is pericholecystic stranding (yellow arrow) and enhancement of the peritoneal lining secondary to peritonitis (yellow arrowhead) resulting from bile leak

In gangrenous cholecystitis, the sonographic Murphy sign is typically absent due to necrotic denervation of the gallbladder wall [1]. The gallbladder wall appears asymmetrically thickened with disruption of the wall, floating linear echogenic intraluminal membranes from sloughed mucosa, and air in the lumen or the wall on US (Fig. 4a). Similar findings are seen on CT and MRI, whereas the disruption of mucosa is seen as an area of decreased or no enhancement on contrast-enhanced CT and MRI (Figs. 4b and 5a). CT is more sensitive and specific in identifying the gas (Fig. 5b). On MRI, gas is noted as a signal void in the gallbladder wall or lumen [18, 33]. As mentioned, gangrenous cholecystitis puts the patients at risk for perforation (Fig. 6), but if the perforation is not recognized it is likely the gallbladder will be less distended on follow-up imaging due to the leaking bile.

**Fig. 7:**
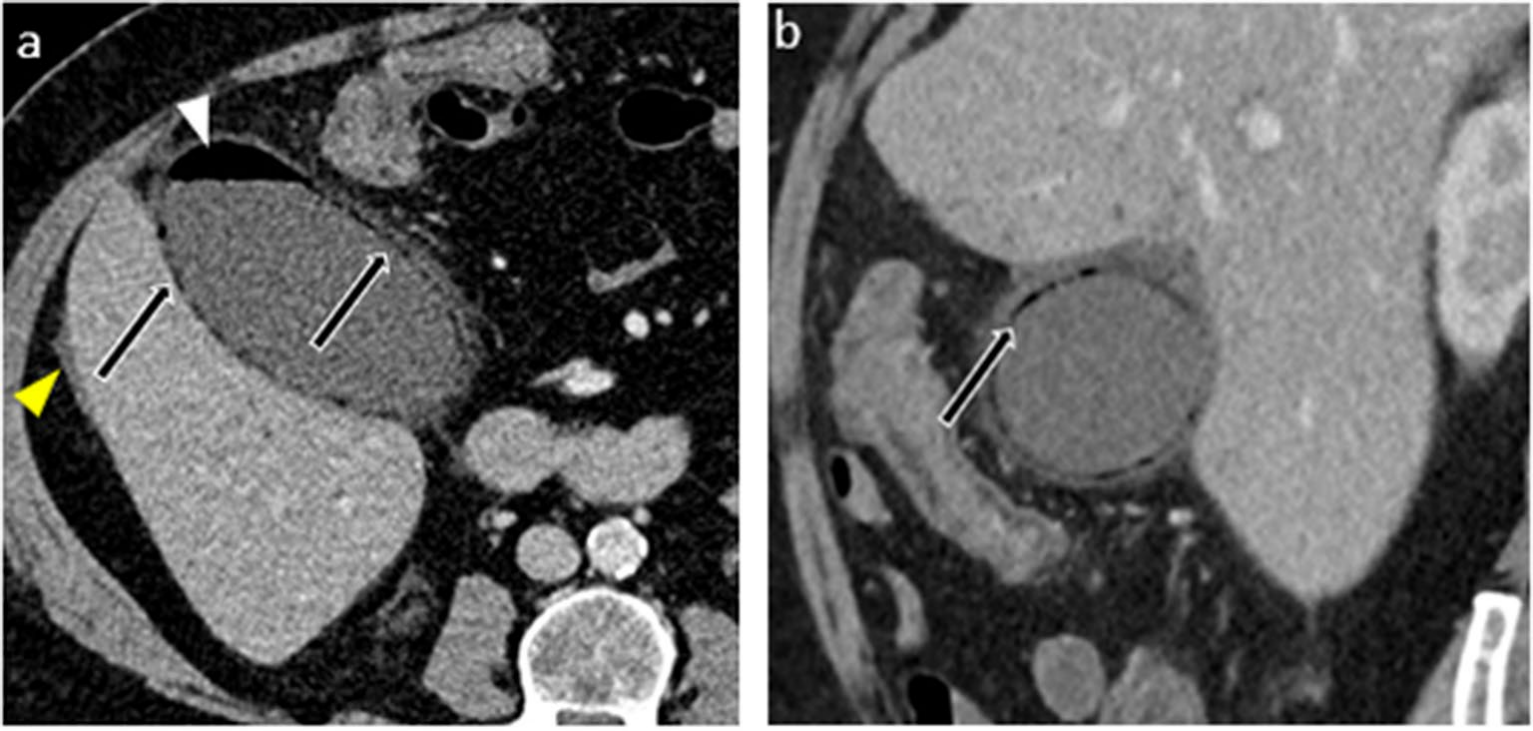
Emphysematous cholecystitis in a 67-year-old female with a history of obesity, diabetes mellitus presents to the ED via walk-in with a chief complaint of abdominal pain, worst in right upper quadrant. Axial (image **a**) and sagittal (image **b**) contrast enhanced CT demonstrates air in the wall (arrows) and in the lumen (arrowhead) of the gallbladder. There is pericholecystic stranding and trace perihepatic fluid (yellow arrowhead)

Emphysematous cholecystitis is seen on US as an echogenic reflection in the gallbladder wall with reverberation artifact (aka “dirty shadowing”). The echogenic reflections correspond to the gas in the wall or lumen of the gallbladder [12]. The gas is seen as hypoattenuating (black) on CT (Fig. 7) and as a signal void or area of artifact on MRI. On MRI, gas either in the lumen or the wall should be interpreted cautiously as calculi, and wall calcification also gives a signal void [33].

Hemorrhagic cholecystitis is another rare complication. In addition to the findings of AAC, blood products are also present as heterogeneous, hyperechoic, and more echogenic than the sludge on US. On CT, the bile is hyperattenuating and may show a fluid-fluid level in the gallbladder from the two different fluid compositions, i.e., bile and blood products. MRI is more sensitive and specific in identifying the blood products in their different stages. Classically, the hemorrhagic bile is seen as T1W hyperintense and T2W hypointense on MRI. The hemorrhagic bile and simple bile give fluid-fluid levels where the former is seen as T2W hypointense lower layer and the simple bile as hyperintense fluid on top. The subacute blood products are hyperintense on both T1 and T2WI [26, 34].

### Chronic acalculous cholecystitis

**Fig. 8:**
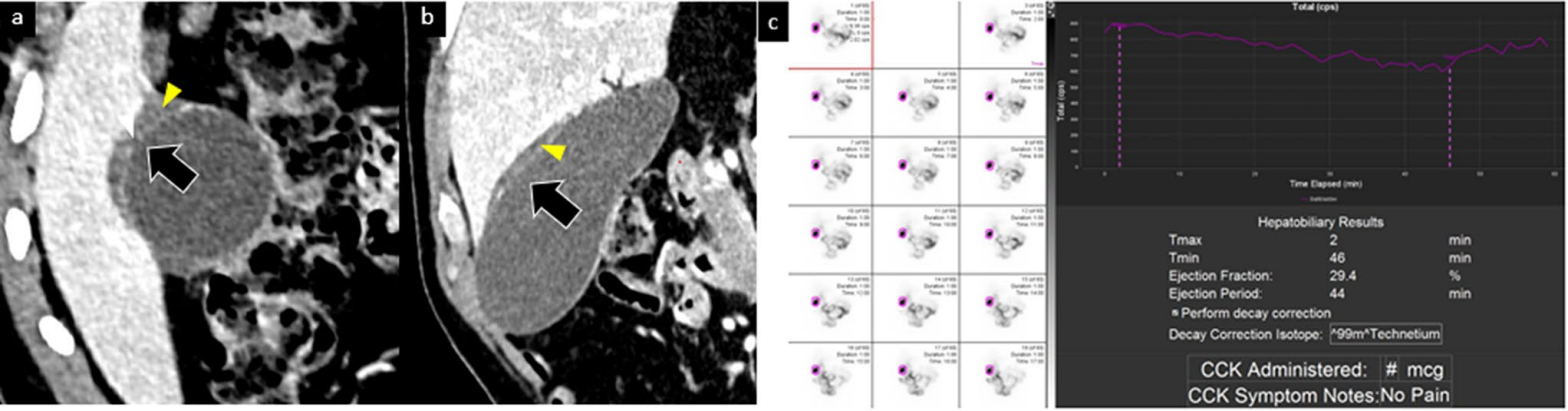
86-year-old man with over three months of right upper quadrant pain. Axial and coronal images of a contrast-enhanced CT (**a**, **b**) shows mildly increased gallbladder wall hyperenhancement with patchy areas hypoenhancement (black arrows) as well as a small amount of pericholecystic fluid. A follow up ultrasound did not show gallstones. A follow up nuclear medicine scintigraphy study (**c**) showed filling of the gallbladder but after a fatty meal a depressed ejection fraction of 29% was found (normal is greater than 33%), compatible with chronic cholecystitis

Patients with CAC may present with nonspecific symptoms such as nausea and vomiting or develop more typical recurrent biliary colic, right upper quadrant pain and jaundice. The risk factors for CAC are not well elucidated and it tends to be a diagnosis of exclusion [35]. Imaging findings in chronic acalculous cholecystitis include gallbladder wall enhancement and thickening, which is a non-specific finding on US, CT, and MRI that requires correlation with clinical symptoms for accurate diagnosis in a patient with intermittent biliary colic [21]. Nuclear medicine studies are often necessary to make the diagnosis and several protocols have been developed in which Sincalide (CCK-8) or a fatty meal can be administered to evaluate gallbladder ejection fraction, with a normal value of > 33% and values less than that compatible with chronic cholecystitis (Fig. 8) [22].

### Therapeutic options

#### Interventional radiology

While surgical intervention is considered the gold standard for the treatment of cholecystitis, patients with AAC are typically critically ill, debilitated, and/or elderly patients often deemed unfit for surgery due to their clinical status and co-morbid conditions. Non-surgical treatment options for biliary decompression play a critical role in this patient population. Image-guided percutaneous cholecystostomy (PC) tube placement, performed by interventional radiologists, represents one such option, with a low risk of adverse events [36, 37] and outcomes that approach the effectiveness of definitive cholecystectomy in the treatment of acalculous cholecystitis.

While cholecystostomy tube placement is typically considered a bridge to definitive surgical intervention in those with calculous cholecystitis, multiple studies have demonstrated that PC may be considered a definitive treatment in the setting of acalculous cholecystitis, without the need for subsequent cholecystectomy [38–40]. Since there are no stones present to cause repeat biliary stasis, as long as gallbladder function recovers after the acute episode acalculous cholecystitis, there should be a relatively low risk of recurrence [41]. This has been demonstrated clinically, with most studies reporting recurrent rates less than 10% after tube removal [42].

#### Percutaneous cholecystostomy procedure

While there are no absolute contraindications to cholecystostomy tube placement, the risk versus benefit of tube placement should be considered on a case-by-case basis, and there are several pre-procedural considerations to ensure safe and effective cholecystostomy tube placement [43]. A safe imaging window must be identified to allow for tube placement without injuring intervening structures, such as colon or small bowel. Coagulopathy is often present in the critically ill and should ideally be corrected to allow for safe tube placement. The presence of ascites is also a relative contraindication, as this predisposes the patient to bile leak and bile peritonitis, peri-catheter leakage, and tube dislodgement.

Technical success rates are typically reported at ≥ 95%. The procedure is typically performed using ultrasound (US) guidance to achieve gallbladder access, followed by fluoroscopic visualization of subsequent steps. US allows for real-time evaluation of the gallbladder and surrounding structures, such as the liver, colon, and diaphragm, allows for color Doppler evaluation of nearby vasculature, and allows the operator to guide needle placement into the gallbladder in real-time. While preferably performed in the interventional suite, the portability of US also allows for bedside placement in those patients too sick to be transported. Once gallbladder access is obtained, bile is aspirated to confirm positioning. A small amount of contrast may be injected to further confirm positioning and position can oftentimes be confirmed using US as well. If injecting contrast, the gallbladder should not be over-distended, as this may cause bacterial translocation, transient bacteria, and sepsis. Diagnostic images should only be obtained on follow-up cholecystograms, once the patient’s condition has improved. Rarely, the procedure may require CT rather than US guidance if there are limited sonographic windows, altered anatomy, or other technical factors requiring use of CT.

A technical considerations regarding cholecystostomy tube placement is the placement via a transhepatic or transperitoneal approach. The transhepatic approach, in which the access tract traverses a small amount of liver parenchyma before entering the bare area of the gallbladder abutting the liver, is associated with decreased risk of bile leak and transcolonic tube placement as well as increased catheter stability, however, this comes with increased risk of hemorrhagic complications associated with traversing hepatic vasculature [41, 44, 45]. The transperitoneal route, in which access to the gallbladder is achieved directly through the abdominal wall, may have a decreased risk of hemorrhage and pleural transgression. It may also be preferred when the transhepatic route is limited by hepatic malignancy or cysts. However, this approach carries increased risk of transgressing bowel, bile leakage, and tube dislodgment [41, 44, 45]. Despite the above considerations, retrospective studies have not shown any significant differences in complications or outcomes between either approach, and choice of access is ultimately operator dependent and should be made on a case-by-case basis.

#### Complications

Most complications related to cholecystostomy tube placement are minor and are comprised of catheter-related issues, such as peri-catheter leakage, dislodgement, or clogging, with dislodgement representing the most common late complication and occurring in 5–15% of patients [46]. Minor bleeding not requiring intervention may also be seen. Major complications are seen in less than 5% of patients and include sepsis, hemorrhage requiring transfusion or intervention, bowel injury, bile leak/bile peritonitis, and hemo/pneumothorax related to pleural transgression [46, 47]. While there is a relatively high 30-day post-procedural mortality rate after cholecystostomy tube placement, reported in the 5–35% range, the large majority of these are related to patient comorbidities in this critically ill population rather than the cholecystostomy tube or procedure itself [46–48]. Procedure-related mortality is reported at less than 0.5% [46].

## Interventional gastroenterology

Although percutaneous cholecystostomy (PC) is a good choice for treatment of AAC, prolonged maintenance of the tube can potentially be uncomfortable, and adverse events such as catheter dislodgement and infection are possible, requiring longer hospital stays and potential reintervention in 25–66% of patients [49–51]. Also, as mentioned, a significant subset of patients with coagulopathy or ascites may not be good candidates for PC.

To address some of these limitations, endoscopic gallbladder drainage procedures have been developed, including endoscopic transpapillary gallbladder drainage (ET-GBD) and endoscopic ultrasound-guided gallbladder drainage (EUS-GBD) [52].

ET-GBD can be performed via a transpapillary approach, placing a plastic stent from the duodenum into the gallbladder via the major papilla and common bile duct. It is preferred if there is a need for simultaneous ERCP (e.g. to treat choledocholithiasis or cholangitis). Limitations to ET-GBD include technical challenges in identifying, cannulating, and navigating a tortuous or strictured cystic duct [53].

In EUS-GBD, the gallbladder is drained with EUS guidance directly into the gastric antrum (cholecystogastrostomy) or duodenum (cholecystoduodenostomy). The procedure used to require transmural puncture between the GI tract and gallbladder with an FNA needle, wire advancement, and tract dilatation to allow placement of double-pigtail plastic stents and/or a self-expandable metal biliary stents over the wire [54]. Fistulous tract leakage and stent migration were potential complications [55]. Lumen-opposing metal stents (LAMS) have now become the stent of choice, due to a simplified electrocautery-enhanced single-step LAMS deployment, which obviates the need for guidewire exchange and fluoroscopy. A prospective multicenter trial of 30 patients with cholecystitis who were not candidates for cholecystectomy found technical success of 93.3%. [56].

PC is the preferred modality for non-invasive GB drainage in patients unable to tolerate general anesthesia since it can be performed under local anesthesia with minimal sedation. For those patients who may undergo future cholecystectomy or liver transplant, ET-GBD is usually preferred since it preserves native biliary anatomy and structural integrity of the GB [57].

### Summary

In summary, acute acalculous cholecystitis (AAC) is an important entity to recognize, especially in critically ill patients. The Tokyo guidelines offer a set of criteria, including radiologic criteria, that can aid in a diagnosis. In addition to making a diagnosis, imaging also guides management by assessing for potential complications (e.g. gangrene and perforation) as well as evaluating for alternative diagnoses. While US serves as the initial modality of choice, nuclear medicine scintigraphy, CT, and MRI provide valuable additional information in complex cases or when US findings are equivocal. The choice of imaging technique is guided by clinical judgment, patient condition, and specific diagnostic needs, highlighting the importance of a multidisciplinary approach in the management of AAC.

Imaging also plays a role in guiding therapeutic interventions, such as in placement of percutaneous cholecystostomy, a valuable and mature therapeutic option for AAC in critically ill patients. Other interventional gastroenterology techniques have also been developed and are of particular use in certain subsets of patients for whom PC is either not feasible or not the ideal therapeutic option.

## Electronic supplementary material

Below is the link to the electronic supplementary material.

Supplementary Material 1

## Data Availability

No datasets were generated or analysed during the current study.
