# Correction: Acalculous cholecystitis– an imaging and therapeutic update

**DOI:** 10.1007/s00261-026-05462-9

**Published:** 2026-06-26

**Authors:** Matthew A. Morgan, Daniel M. DePietro, Debra S. Whorms, Austin R. Pantel, Dhakshinamoorthy Ganeshan, Inessa A. Goldman, Julie Yang, Rachita Khot

**Affiliations:** 1 University of Pennsylvania Health System, Philadelphia, USA; 2 The University of Texas MD Anderson Cancer Center, Houston, USA; 3 Rutgers New Jersey Medical School, Newark, USA; 4 Icahn School of Medicine at Mount Sinai, New York, USA; 5 University of Virginia Health, Charlottesville, USA

**Correction to: Abdominal Radiology (2025) 50:2881–2891**

**https://doi.org/10.1007/s00261-024-04691-0**

In this article, instead of Fig. 7, Fig. 6 was incorrectly used twice and in Fig. 8 image (c) is missing. For completeness and transparency, the old incorrect and the correct Fig. 7 and 8 are displayed below. Also, on page 6, the reference to image 5b is missing and it should appear as “CT is more sensitive and specific in identifying the gas (Fig. 5b).”

Incorrect version of Fig. 7:

**Fig. 7:**
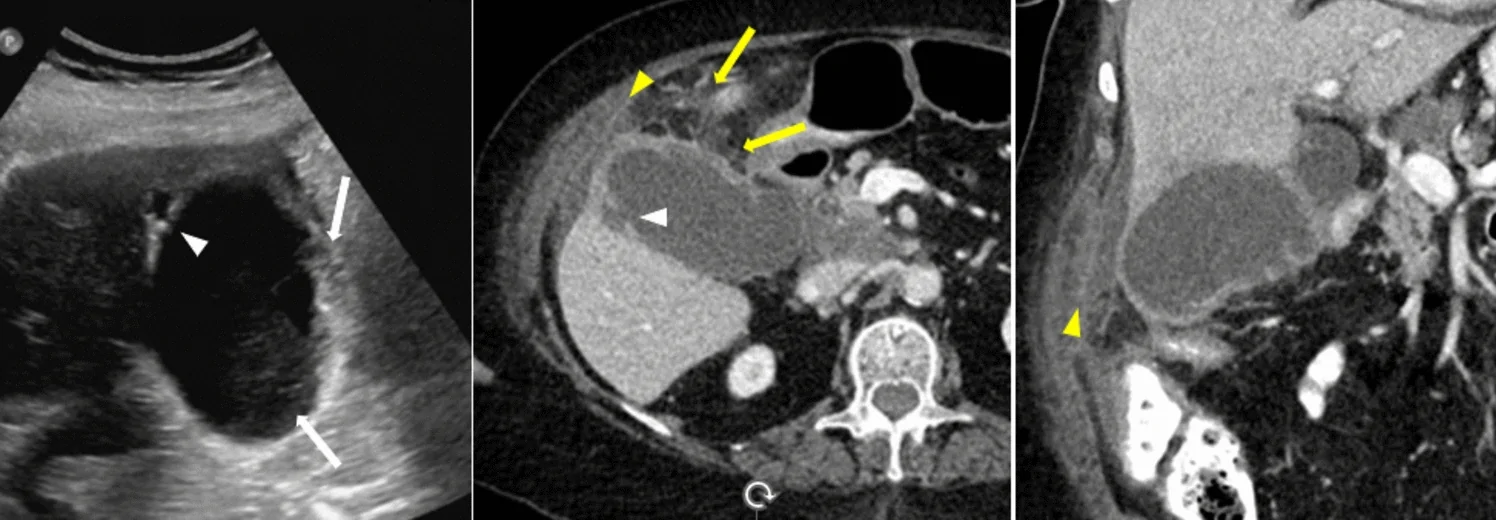
Emphysematous cholecystitis in a 67-year-old female with a history of obesity and diabetes mellitus presents to the ED via walk-in with a chief complaint of abdominal pain, worst in right upper quadrant. Axial (image **a**) and sagittal (image **b**) contrast enhanced CT demonstrates gas in the wall (arrows) and in the lumen (arrowhead) of the gallbladder. There is pericholecystic stranding and trace perihepatic fluid (yellow arrowhead)

Correct version of Fig. 7:

**Fig. 7:**
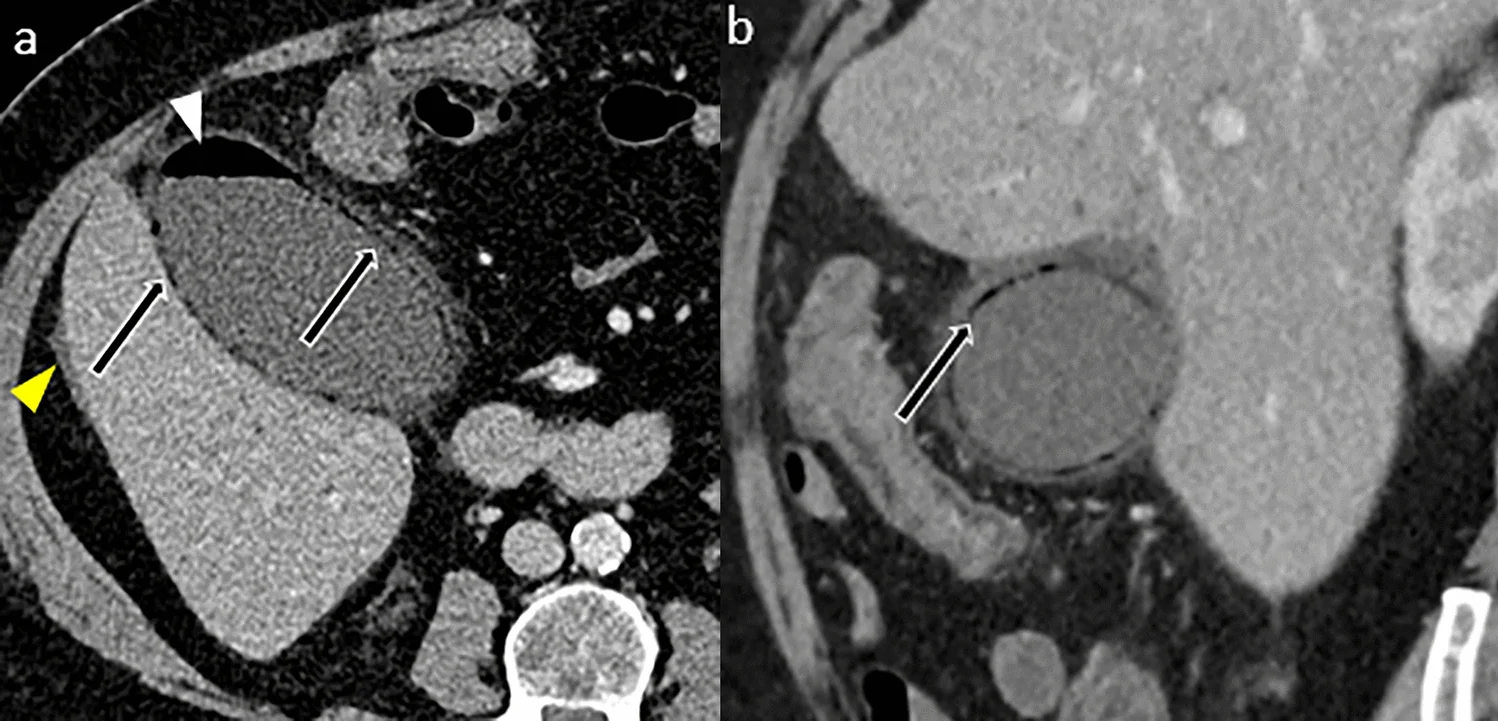
Emphysematous cholecystitis in a 67-year-old female with a history of obesity and diabetes mellitus presents to the ED via walk-in with a chief complaint of abdominal pain, worst in right upper quadrant. Axial (image **a**) and sagittal (image **b**) contrast enhanced CT demonstrates gas in the wall (arrows) and in the lumen (arrowhead) of the gallbladder. There is pericholecystic stranding and trace perihepatic fluid (yellow arrowhead)

Incorrect version of Fig. 8:

**Fig. 8:**
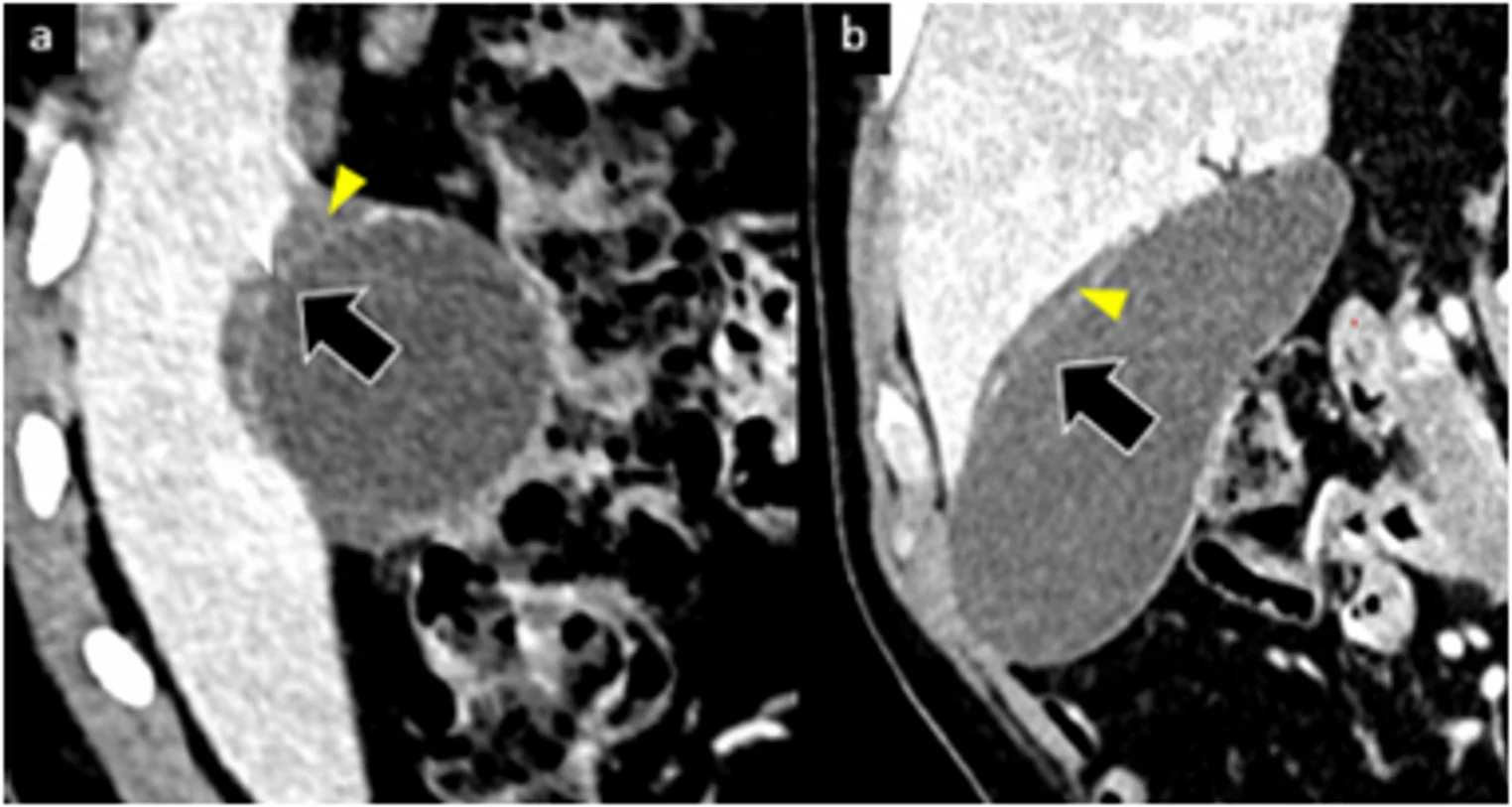
86-year-old man with over three months of right upper quadrant pain. Axial and coronal images of a contrast-enhanced CT (**a**, **b**) shows mildly increased gallbladder wall hyperenhancement with patchy areas of hypoenhancement (black arrows) as well as a small amount of pericholecystic fluid. A follow up ultrasound did not show gallstones. A follow up nuclear medicine scintigraphy study (**c**) showed filling of the gallbladder but after a fatty meal a depressed ejection fraction of 29% was found (normal is greater than 33%), compatible with chronic cholecystitis

Correct version of Fig. 8:

**Fig. 8:**
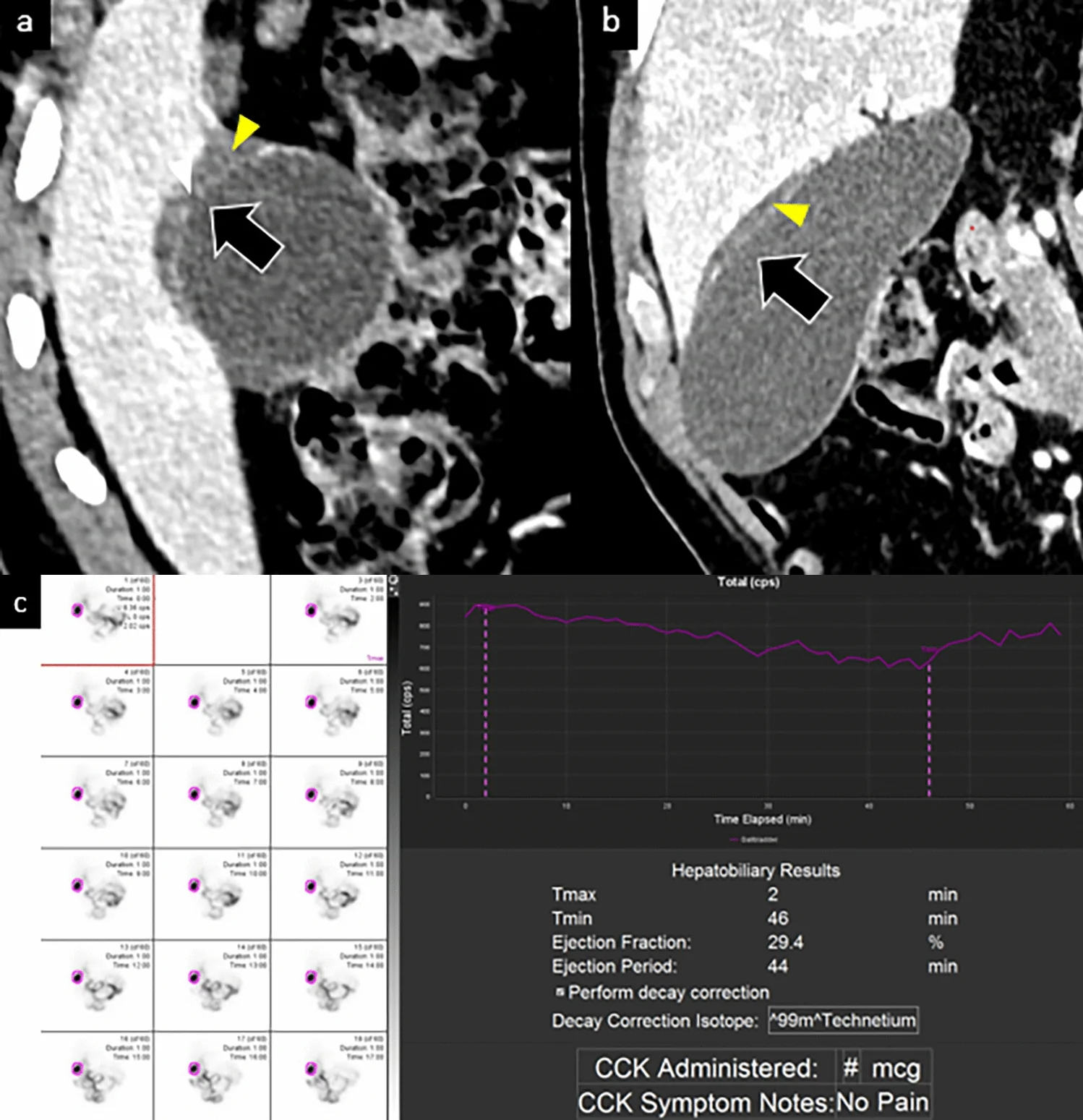
86-year-old man with over three months of right upper quadrant pain. Axial and coronal images of a contrast-enhanced CT (**a**, **b**) shows mildly increased gallbladder wall hyperenhancement with patchy areas of hypoenhancement (black arrows) as well as a small amount of pericholecystic fluid. A follow up ultrasound did not show gallstones. A follow up nuclear medicine scintigraphy study (**c**) showed filling of the gallbladder but after a fatty meal a depressed ejection fraction of 29% was found (normal is greater than 33%), compatible with chronic cholecystitis

The original article has been corrected.

